# Post-cholecystectomy Hepatic Subcapsular Biloma: A Detailed Case Study on Presentation and Management

**DOI:** 10.7759/cureus.55966

**Published:** 2024-03-11

**Authors:** Mena Louis, Bradley Kuhn, Nicole Redenius

**Affiliations:** 1 General Surgery, Northeast Georgia Medical Center, Gainesville, USA; 2 Trauma and Acute Care Surgery, Northeast Georgia Medical Center, Gainesville, USA

**Keywords:** post-cholecystectomy complication, minimally invasive management, percutaneous drainage, laparoscopic cholecystectomy, hepatic subcapsular biloma

## Abstract

Hepatic subcapsular biloma is a rare but significant complication following laparoscopic cholecystectomy, characterized by the accumulation of bile beneath the hepatic capsule. Despite its infrequency, recognizing this condition is crucial due to its potential for significant morbidity. This report aims to elucidate the presentation, diagnosis, and management of this complication to enhance clinical outcomes. We present the case of a 59-year-old male with a complex medical history including atrial fibrillation, heart failure with preserved ejection fraction, myocardial infarction, chronic obstructive pulmonary disease, hypertension, and alcohol abuse. The patient presented with acute cholecystitis and underwent an uncomplicated laparoscopic cholecystectomy. Postoperatively, he developed right upper quadrant abdominal pain and nausea, leading to the diagnosis of a hepatic subcapsular biloma. The biloma was managed successfully with percutaneous drainage, illustrating a rare complication managed effectively without the need for endoscopic retrograde cholangiopancreatography (ERCP). This case illustrates the need for heightened awareness and swift imaging to diagnose hepatic subcapsular biloma effectively. The management of this patient demonstrates the effectiveness of percutaneous drainage in resolving bilomas and avoiding more invasive procedures such as ERCP. This case adds to the limited literature on the management of post-cholecystectomy hepatic subcapsular biloma and emphasizes the importance of considering this diagnosis in similar clinical scenarios. In conclusion, hepatic subcapsular biloma is a rare complication post-cholecystectomy that requires early recognition and intervention. This case contributes to the body of knowledge, emphasizing the role of imaging in diagnosis and the effectiveness of minimally invasive management strategies. It highlights the educational value of recognizing early postoperative complications, thereby enhancing patient safety and care.

## Introduction

Laparoscopic cholecystectomy is a common and generally safe surgical procedure for the treatment of symptomatic cholelithiasis and gallbladder diseases including conditions such as acute cholecystitis, chronic cholecystitis, gallbladder polyps, and biliary dyskinesia [1-3]. It has a well-established profile for reducing postoperative pain, shortening hospital stays, and enabling quicker returns to normal activities compared to open cholecystectomy [4,5]. Despite these advantages, it is not without complications; among these, bile duct injuries and subsequent bile leaks represent significant postoperative challenges, with an incidence rate reported between 0.3% and 0.6% in large series [6-8]. Bilomas, encapsulated collections of bile outside the biliary tree within the abdominal cavity, are a relatively rare but serious complication following laparoscopic cholecystectomy and endoscopic retrograde cholangiopancreatography (ERCP) [9,10].

Introduced in 1979 by Gould and Patel, the term "biloma" was initially used to describe loculated collections of bile outside the biliary tree [11]. These collections can occur intrahepatically or extrahepatically and result from iatrogenic injuries, from traumatic events, or spontaneously due to biliary tree rupture [12,13]. Despite their uncommon occurrence, bilomas, particularly hepatic subcapsular bilomas, pose a significant risk due to their potential to lead to severe complications such as infection, abscess formation, and peritonitis if not diagnosed and managed promptly [14-16].

The pathophysiology behind biloma formation involves the disruption of the biliary tree's integrity, leading to bile leakage into the abdominal cavity or subcapsular space of the liver [17]. This disruption can be a consequence of surgical manipulation, accidental injury during procedures, or high-pressure events such as those induced during ERCP. The clinical presentation of bilomas is variable, often including symptoms like abdominal pain, nausea, vomiting, and jaundice, complicating the diagnostic process [18]. Imaging studies, including ultrasound and computed tomography (CT), play a crucial role in the diagnosis and management of bilomas, guiding therapeutic interventions like percutaneous drainage [19].

This article aims to highlight the rare complication of hepatic subcapsular biloma following laparoscopic cholecystectomy, emphasizing the importance of early diagnosis and management. Through a detailed case presentation, we will explore the clinical journey of a patient from presentation through to diagnosis and successful management, providing insights into best practices and stressing the need for vigilance in postoperative care to mitigate the risk of this potentially life-threatening complication.

## Case presentation

**Figure 1:**
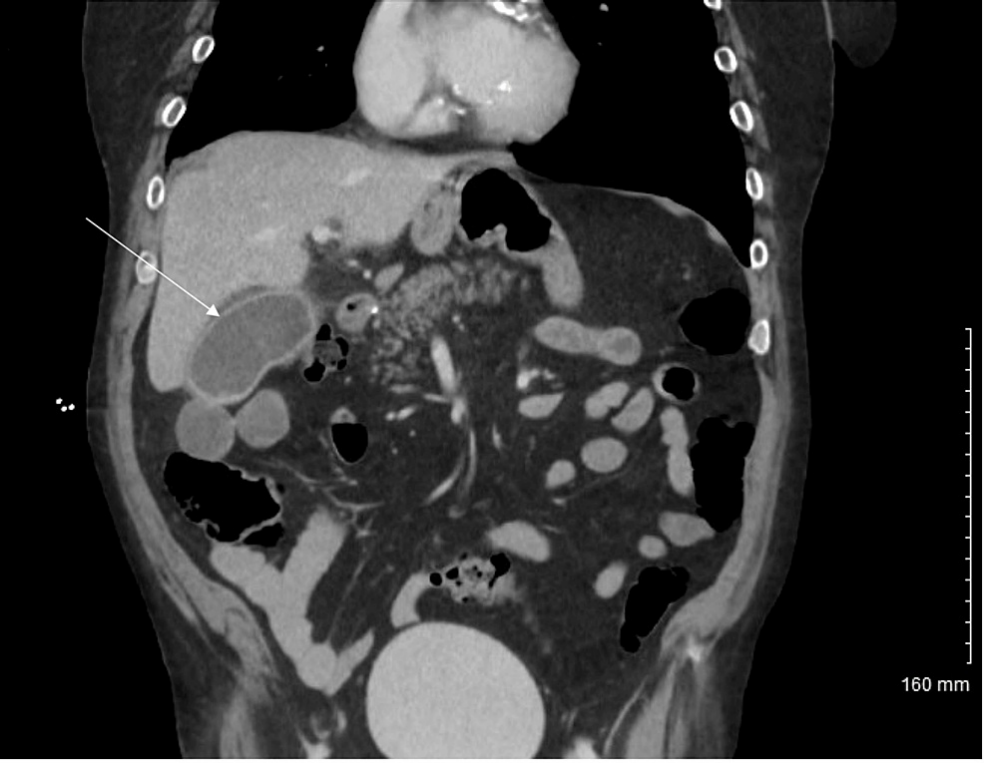
CT of the abdomen and pelvis coronal section showing distended gallbladder with mucosal hyperenhancement and trace pericholecystic fat stranding (white arrow). CT: computed tomography

A 59-year-old male with a complex medical history, including atrial fibrillation managed with apixaban, heart failure with preserved ejection fraction, a previous myocardial infarction treated with coronary artery bypass grafting six months prior, chronic obstructive pulmonary disease, hypertension, and a history of alcohol abuse, presented with acute right upper quadrant abdominal pain accompanied by nausea and vomiting. His past surgical history was notable for a partial gastrectomy due to a perforated gastric ulcer. Physical examination revealed tenderness in the right upper quadrant with a positive Murphy's sign, suggesting acute cholecystitis. Laboratory findings are shown in Table 1. Imaging studies, including a CT of the abdomen and pelvis (Figure 1) and an ultrasound of the right upper quadrant, demonstrated a distended gallbladder with thickening of the gallbladder wall and pericholecystic fluid, confirming the diagnosis of acute cholecystitis.

**Table 1:** Lab values on initial presentation. BUN: blood urea nitrogen

Lab value	Result	Reference/range
White blood cell count	9.0	4.8-10.8 K/uL
Hemoglobin	9.5	12.0-16.0 g/dL
Platelets	300	130-400 K/uL
BUN	10.0	5.0-23.0 mg/dL
Creatinine	0.76	0.60-1.00 mg/dL
Total bilirubin	1.10	0.00-1.00 mg/dL

The patient was planned for cholecystectomy after a 48-hour discontinuation of apixaban and was started on piperacillin-tazobactam. The laparoscopic cholecystectomy was performed without event; the procedure involved the open Hasson technique for abdominal entry, placement of subxiphoid and right upper quadrant trocars under direct vision, and management of significant omental adhesions to the gallbladder through blunt dissection. Due to gallbladder distension, needle decompression was performed, followed by the dissection of the infundibulum, cystic duct, and cystic artery. The gallbladder was removed after achieving a critical view of safety and ensuring hemostasis. Postoperatively, apixaban was resumed on the first postoperative day, and despite a prolonged stay due to postoperative ileus, the patient was discharged on the sixth postoperative day.

**Figure 2:**
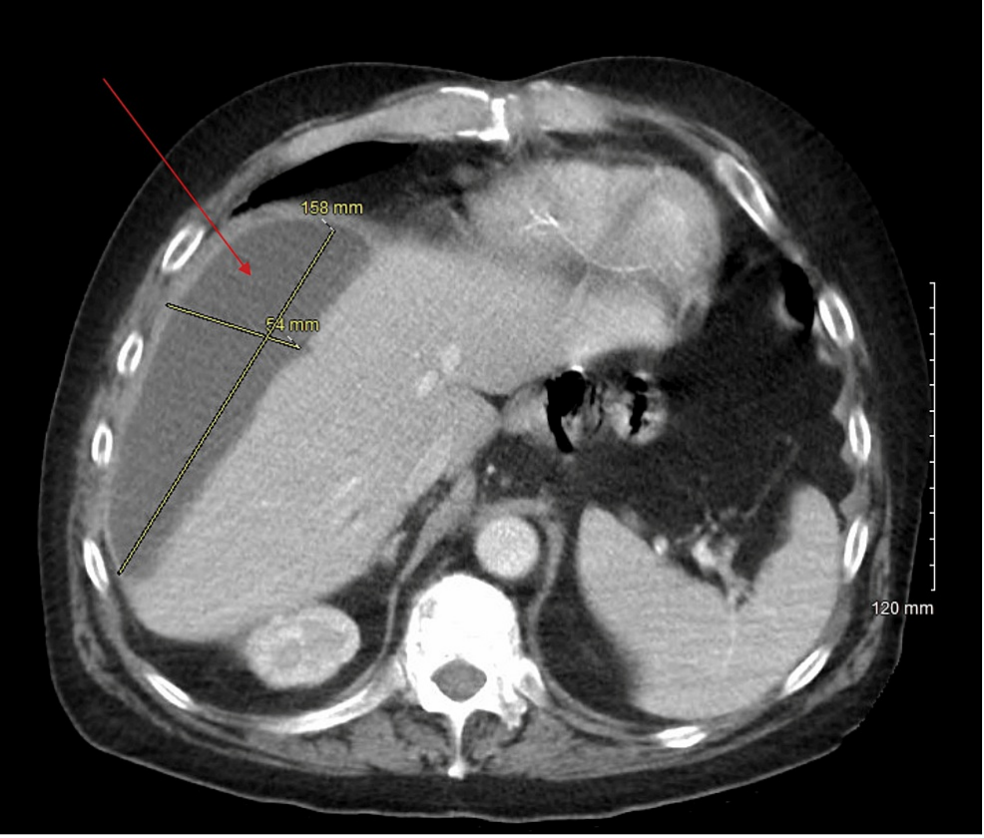
Axial CT scan of the abdomen showing subcapsular fluid collection in the right perihepatic space (red arrow). CT: computed tomography

**Figure 3:**
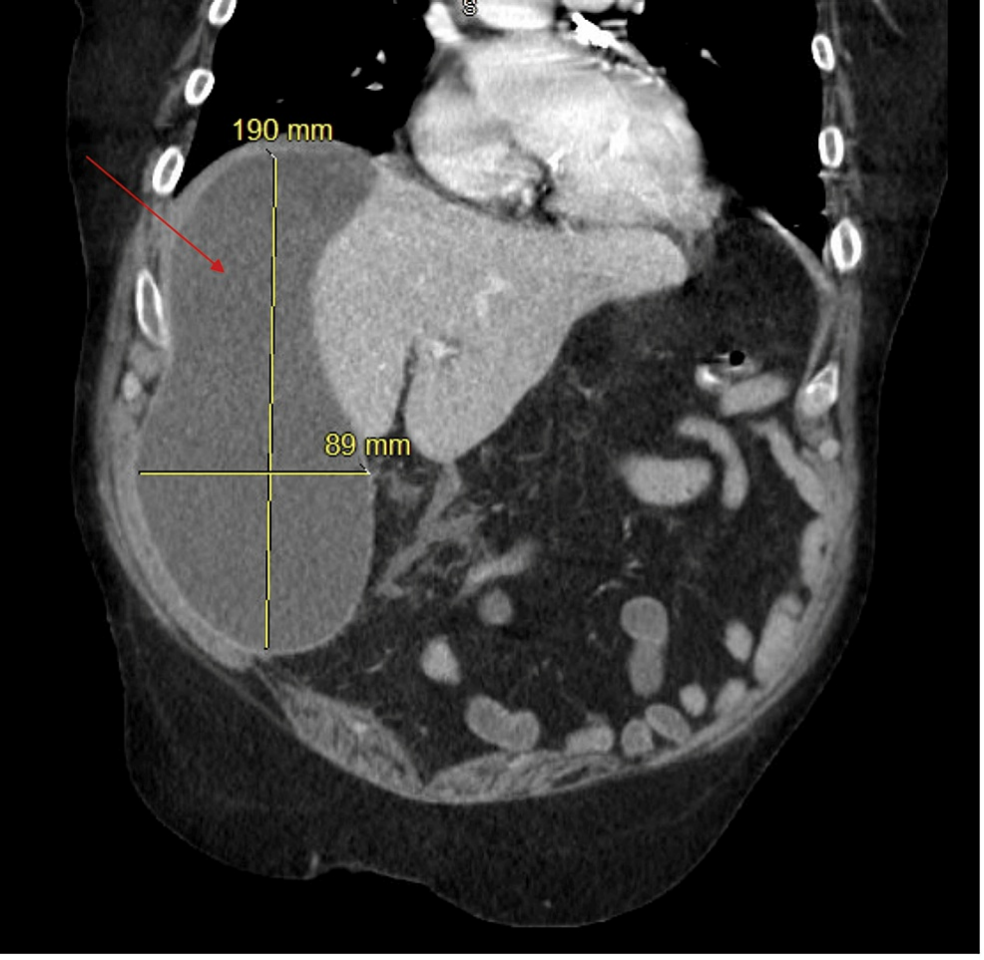
Coronal CT of the abdomen with subcapsular hepatic biloma measuring 19 x 8.9 cm. CT: computed tomography

**Figure 4:**
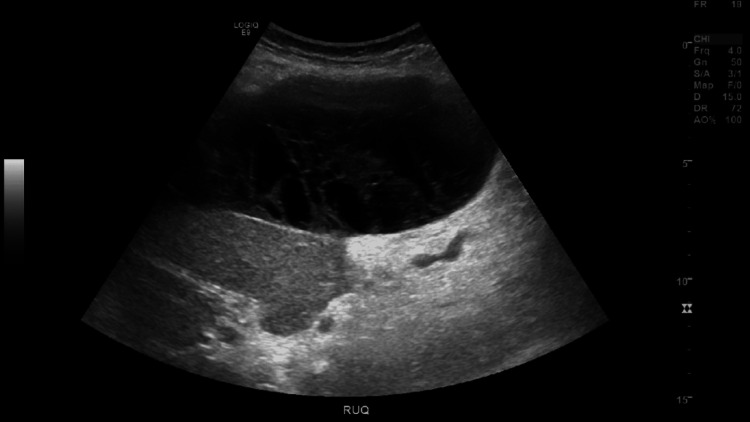
RUQ ultrasound showing hepatic subcapsular collection with multiple loculations. RUQ: right upper quadrant

However, on the 13th postoperative day, the patient returned to the emergency department with worsening abdominal pain and nausea. Laboratory tests including liver function were within normal limits, except for a slightly elevated white blood cell count of 12.8. A repeat CT of the abdomen and pelvis revealed a significant subcapsular fluid collection in the right perihepatic space, suggestive of an abscess or biloma, a complication of the recent cholecystectomy (Figure 2, Figure 3). An ultrasound-guided placement of a right upper quadrant biloma drainage catheter was performed, revealing multiple septations and loculations within the fluid collection (Figure 4). Despite initial drainage, a follow-up CT scan indicated a marginal increase in the size of the collection, necessitating up-sizing of the abdominal drain and additional drainage catheter placement. Throughout this hospital stay, the patient's liver function tests remained stable.

This case explores the diagnosis and management of hepatic subcapsular bilomas post-cholecystectomy, especially in patients with significant comorbidities. The absence of difficult dissection or pressurization of the biliary tree during surgery, coupled with the effective management through percutaneous drainage without the need for ERCP, highlights the importance of early detection and intervention in the successful resolution of this uncommon complication.

## Discussion

The occurrence of a hepatic subcapsular biloma following laparoscopic cholecystectomy, as presented in this case, is a rare but significant postoperative complication requiring prompt recognition and management [18]. 

The etiology of hepatic subcapsular biloma primarily involves iatrogenic injury to the biliary tract, which can occur during laparoscopic cholecystectomy [12,16]. Despite the procedure's routine nature and its reputation for safety, the risk of biliary injury persists, potentially leading to bile leakage and biloma formation [4]. In this case, the absence of difficult dissection or notable pressurization of the biliary tree during surgery suggests that even uneventful procedures can result in complications, possibly due to unrecognized micro-injuries to the biliary ducts or the high-pressure irrigation.

Diagnostic challenges arise from the biloma's insidious presentation and the variability of symptoms. As demonstrated, the patient returned with worsening abdominal pain and nausea, common postoperative complaints that may not immediately suggest a biloma. The role of imaging, particularly CT and ultrasound, is crucial in diagnosing subcapsular biloma, as these modalities can identify fluid collections distinct from other postoperative phenomena like hematomas or abscesses. This case emphasizes the importance of considering biloma in the differential diagnosis of post-cholecystectomy patients presenting with abdominal pain, even when liver function tests are normal.

The fact that ultrasound detected multiple loculations not visible on CT imaging serves as a compelling argument for adopting a multifaceted imaging approach in the diagnosis of hepatic subcapsular biloma. This finding emphasizes the importance of utilizing a combination of imaging modalities to capture the complete scope of postoperative complications.

Management strategies for hepatic subcapsular biloma have evolved, with a growing preference for minimally invasive techniques [17]. Percutaneous drainage, as employed in this case, represents a cornerstone of biloma management, avoiding the need for more invasive procedures like ERCP or surgical revision. The successful management of this patient with percutaneous drainage alone, without ERCP, adds to the growing body of evidence supporting this approach. It highlights the necessity of a tailored management strategy that considers the patient's overall condition, the size and characteristics of the biloma, and the presence of underlying biliary tree pathology.

## Conclusions

Hepatic subcapsular biloma is a condition characterized by the accumulation of bile beneath the liver's capsule, following a laparoscopic cholecystectomy. The formation of a biloma is often attributed to bile leakage, potentially due to surgical manipulation or micro-injuries to the biliary ducts. This case, effectively managed through percutaneous drainage, highlights an important management strategy, demonstrating that not all bilomas require ERCP for resolution.
